# Book Review: Algal Green Chemistry Recent Progress in Biotechnology

**DOI:** 10.3389/fbioe.2018.00096

**Published:** 2018-07-13

**Authors:** Sirisha L. Vavilala, Siddhesh B. Ghag, Jacinta S. D'Souza

**Affiliations:** School of Biological Sciences, UM-DAE Centre for Excellence in Basic Sciences, Kalina campus, Mumbai, India

**Keywords:** algae, cytoprotectants, bioproducts, therapeutics, pigments, biotechnology

An apt for the current issue, the book consists of 14 chapters that provide interesting scientific insights into the vastly rich algal resource. While the book is unstructured, we have divided the review into four scientifically relevant sections (i) Cytoprotectants, (ii) pigments/therapeutics, (iii) bioproducts, and (iv) biomass production.

Chapter one introduces algal osmoprotectants, highlighting the diverse research performed with salinity and its impact on photosynthetic organisms; it specifically focuses on saccharides, glycine betaine, glycerols and dimethylsulfoniopropionate and the accumulation of the last three molecules under abiotic stress. For glycine betaine, the emphasis is on gene expression and enzymatic regulation, while for dimethylsulfoniopropionate the “omics” approach to identify biosynthetic enzymes is elaborated. Further, it expands on the role of Mycosporines and Mycosporine-like amino acids (MAAs) as sunscreen, osmoprotectant, and antioxidants. In chapter two, UV-induced effects on various organisms and the search for algal photoprotectants is discussed. It emphasizes on the role, occurrence, genetic/environmental regulation, and biosynthesis of algal sun protectants (glycosylated MAAs and Scytonemins); further focusing on MAAs that is again described in chapter five. Among cyanobacterial MAAs, the chapter emphasizes upon asterina-330, palythine, palythinol, euhalothece-362, and mycosporine-2-glycine and explains the novel MAAs that exist in algae. It concludes with how UV photoprotectants from nature remain a key exploration area.

Chapter five deals with natural antioxidants with a therapeutic perspective. It begins by highlighting the effect of ROS accumulation on biomolecules; discussing further on oxidative stress-induced mitochondrial irregularities. The occurrence, structure, mode of action, and putative roles in therapeutics of algae-based antioxidants such as phycobiliproteins, phlorotannins, carotenoids, sulfated polysaccharides, Scytonemins, and MAAs is justified. The authors suggest the need for further research to identify high bioactivity molecules. Chapter four deals with the emerging area of nutraceuticals. While microalgae have been a research area for decades, it has only recently served as source of chemicals/pharmaceuticals. It focuses on a variety of nutrients in algae including polyunsaturated fatty acids, vitamins, polysaccharides, and proteins; but, expands on the various categories of polyunsaturated fatty acids, structural chemistry, algal sources, and particularly the health benefits of Omega-3 fatty acids. The authors highlight the types, structural chemistry, algal sources and nutraceutical/biotechnological applications of β-carotene, astaxanthin, and lutein, with a brief mention of the industrial and health applications of algal pigments, vitamins, polysaccharides, Mycosporine and MAAs and bioactive peptides. It concludes with the benefits of nutritionally important algae such as *Haematococcus pluvialis, Chlorella, Spirulina, Nannochloropsis*. In chapter seven, the authors expand on the factors that influence carotenogenesis like light intensity, temperature, salinity, and nutrient limitation and a description of pigment extraction, applications, and future prospects. In chapter nine, the authors organized the chemical structures, absorbance maxima, and distribution of pigments in a tabular format. It also covers commercialized algal pigments used as skin ointments and cosmetics; however, the application of these pigments in dye/textile industry and as food colorants remains unaddressed. Chapter eight describes the synthesis and catabolism of γ-amino butyric acid and polyamines that accumulate in stress-exposed cells. Accumulation of γ-amino butyric acid in cyanobacteria can be correlated with the nitrogen/carbon source and also supplementation with polyamines (putrescine and spermidine) in the growth medium; the latter accumulate with stress. Variations in the enzymes that synthesize polyamines in cyanobacteria have been observed. Chapter 12 summarizes the occurrence, metabolism and biological significance of polyamines in microalgae and sea grasses. It compiles the investigation of molecular mechanisms of polyamines of macroalgae under stress. However, a comparison between polyamines in macroalgae and higher plants could have been more insightful.

Chapter six discusses several routes ranging from the direct or derivative-based use of microalgal biomass or engineering microalgae to make bioplastics. While the challenge lies in the field of harvesting, innovative cultivation techniques have contributed to its scientific progress. An excellent compilation of the ratios that make use of microalgae producing PHAs blended with petrochemical plastics is provided as a ready-reckoner. Chapter 10 deals with the application of algal species as a biofertilizer that are capable of reclaiming sodic soils and improving the soil microflora. Engineered cyanobacteria have been developed with novel transgenes for upgrading biofertilizer technology. However, translating from lab to field is challenging but worth investing. Chapter 14 deals with algal biofilms that have been visualized as a stress-responsive physiology and a nuisance to the environment. These are now exploited for waste water treatment, nutrient sequestration, as biofertilizers, in the form of biological soil crusts and in the production of biofuels. The authors admit that a thorough understanding of algal biofilms is imminent, a lot needs to be done. Bioenergy derived from algal biomass has been an intensively pursued field and chapter 11 provides an exhaustive overview. It describes ways of optimizing the reactor parameters for increased biomass production. Before standardizing, mathematical simulations are a good starting point. In particular, three models have been put forth for light availability. Added to this issue is, the removal of spent medium and O_2_ which inhibit algal growth. Several downstream processes have been provided using single/mixed algal cultures and the challenge in harvesting and extracting the bio-oil remains. It gives the reader a comprehensive account of bio-oil production. Chapter three deals with the proteome-based approach in *Spirulina* for bioproducts production. The authors depict the use of multidiscipline for the implementation of knowledge-to-process implying the need for basic knowledge in biochemical synthesis and regulation in understanding cellular functioning.

Finally, chapter 13 concerns the strategies used in the production of microalgal biomass. Considering their diverse forms, and the potential they exhibit, the nutrients required, growth conditions and the use of appropriate strain is proportionate to the production of biomass; this being equally diverse and heterogeneous.

The research areas covered in this book are rapidly expanding. The authors have comprehensively compiled literature on algal compounds and their applications with depth and clarity. It is handy for students, researchers and industrialists working in the field of algal bioactive compounds. It highlights both recent and past advances in the field presenting the challenges and prospects of translating laboratory research for commercial application.

## Author contributions

All authors listed have made a substantial, direct and intellectual contribution to the work, and approved it for publication.

### Conflict of interest statement

The authors declare that the research was conducted in the absence of any commercial or financial relationships that could be construed as a potential conflict of interest.

